# New Low-Frame-Rate Compensating Pixel Circuit Based on Low-Temperature Poly-Si and Oxide TFTs for High-Pixel-Density Portable AMOLED Displays

**DOI:** 10.3390/mi12121514

**Published:** 2021-12-05

**Authors:** Ching-Lin Fan, Wei-Yu Lin, Chun-Yuan Chen

**Affiliations:** 1 Graduate Institute of Electro-Optical Engineering, National Taiwan University of Science and Technology, No. 43, Sec. 4, Keelung Rd., Da’an Dist., Taipei City 106, Taiwan; d11019001@mail.ntust.edu.tw (W.-Y.L.); d10519002@mail.ntust.edu.tw (C.-Y.C.); 2 Department of Electronic and Computer Engineering, National Taiwan University of Science and Technology, No. 43, Sec. 4, Keelung Rd., Da’an Dist., Taipei City 106, Taiwan

**Keywords:** active-matrix organic light-emitting diode (AMOLED), low frame rate, low-temperature polycrystalline silicon and oxide (LTPO), compensating pixel circuit, portable displays

## Abstract

A new low-frame-rate active-matrix organic light-emitting diode (AMOLED) pixel circuit with low-temperature poly-Si and oxide (LTPO) thin-film transistors (TFTs) for portable displays with high pixel density is reported. The proposed pixel circuit has the excellent ability to compensate for the threshold voltage variation of the driving TFT (ΔV_TH_DTFT_). By the results of simulation based on a fabricated LTPS TFT and a-IZTO TFT, we found that the error rates of the OLED current were all lower than 2.71% over the range of input data voltages when ΔV_TH_DTFT_ = ±0.33 V, and a low frame rate of 1 Hz could be achieved with no flicker phenomenon. Moreover, with only one capacitor and two signal lines in the pixel circuit, a high pixel density and narrow bezel are expected to be realized. We revealed that the proposed 7T1C pixel circuit with low driving voltage and low frame rate is suitable for portable displays.

## 1. Introduction

In recent years, active-matrix organic light-emitting diode (AMOLED) displays have been commonly used in high-end portable displays, because they exhibit various advantages, such as shorter response time, higher contrast ratio, and thinner thickness compared with conventional liquid crystal displays (LCDs) [1,2]. Although the new technologies of Mini-LED and Micro-LED are expected to be the next-generation displays, they suffer from the issues including massive transfer yield and defect repair, which makes it hard to replace the OLED displays in the market currently [3,4].

The backplane technology of low-temperature poly-Si (LTPS) thin-film transistors (TFTs) are widely used in pixel circuits for portable AMOLED displays, since it has excellent current capability [5,6,7]. However, it has some demerits, such as nonuniformity of threshold voltage (V_TH_) and high manufacturing costs [8,9]. Compared with LTPS TFTs, oxide TFTs afford many advantages, including excellent uniformity, favorable stability, low current leakage, and low cost; therefore, large-sized AMOLED displays have adopted them as driving devices [10,11]. Recently, the combined use of LTPS and oxide TFTs, called LTPO, in portable AMOLED displays, has attracted much attention, because it effectively reduces power consumption [12].

Several compensation pixel circuits using LTPS or oxide TFTs as driving devices have been reported to solve issues of threshold voltage variation of the driving TFT (ΔV_TH_DTFT_) [13,14,15,16,17]. Apple Inc. [14] developed the first LTPO compensation pixel circuit for an AMOLED display, in which pixels could be operated at 1 Hz with no flicker phenomenon. However, the pixel density was not high enough for use in high-resolution displays, because it had too many components, including six TFTs, one capacitor, and seven lines. Lin et al. [15] reported a 9T2C pixel circuit using leakage prevention scheme to make AMOLED displays be driven at a low frame rate; however, this circuit was difficult to fabricate because it included nine TFTs, two capacitors, and six lines. Portable AMOLED displays must have low power consumption and high pixel density. To achieve low power consumption, low voltage driving, and a low reset current, are the two main issues. To realize these two objectives, LTPO backplane technology is a suitable candidate. By using LTPS TFT as the driving TFT in the pixel circuit, low voltage driving can be achieved, owing to its high current driving capability. Furthermore, owing to the use of an oxide TFT as the switching TFT, the low current leakage of the oxide TFT confers the pixel circuit with a good ability to hold the voltage; therefore, a small capacitor can be adopted in the pixel circuit, and a low frame rate can be achieved. Moreover, to realize a narrow bezel by integrating a gate on array (GOA), the control signals should be as simple as possible [18]. Furthermore, the number of signal line in a pixel must be small enough for realizing a small pixel size and, in turn, a high pixel density.

This study proposed a low-power-consumption pixel circuit containing two signal lines, and utilizing LTPO TFTs to compensate for the threshold voltage variation of the driving TFTs (ΔV_TH_DTFT_). The simulation results showed that the error rates of the driving current were all less than 2.71% when the V_TH_ variation of driving TFTs was ±0.33 V. Moreover, with low driving voltage of 5 V, and the ability to be driven at a low frame rate of 1 Hz, we believe that the proposed 7T1C pixel circuit with two signal lines is suitable for portable applications.

## 2. Proposed Pixel Circuit Operation

**Figure 1:**
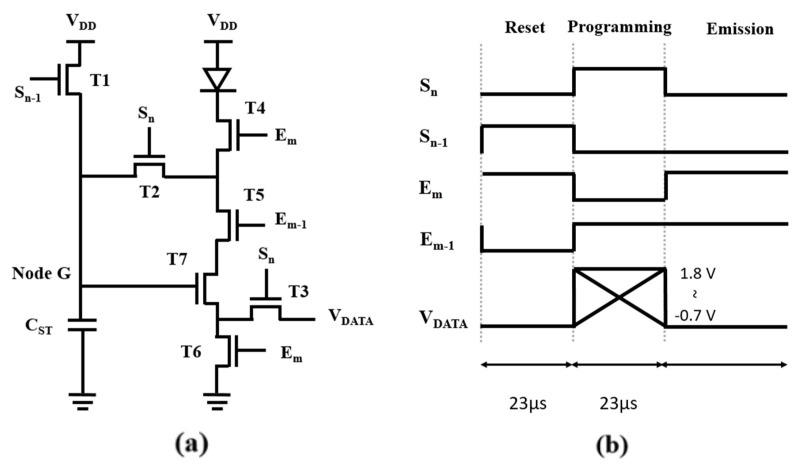
Proposed pixel circuit. (**a**) Circuit schematic. (**b**) Operation timing diagram.

**Figure 2:**
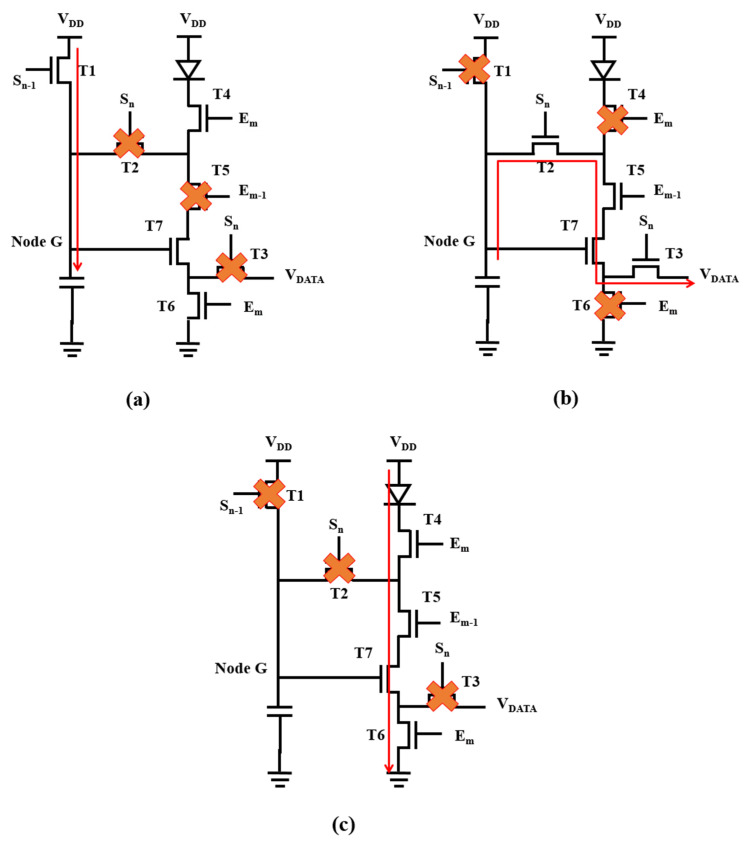
Schematic of circuit operation in (**a**) reset period; (**b**) programming; (**c**) emission period.

Figure 1a shows the proposed pixel circuit. It comprises three switching oxide TFTs (T1–T3), three switching LTPS TFTs (T4–T6), one driving LTPS TFT (T7), and one storage capacitor (C_ST_). The channel length [width (W)/length (L)] of all TFTs is set to 3 μm/3 μm, and the capacitance of C_ST_ is 0.2 pF. The voltage of S_n_ is in the range of −1 to 6.5 V, and the voltage E_m_ is in the range of −3 to 6.5 V. V_DD_ is set to 5 V, while V_SS_ is set to 0 V, respectively. The threshold voltage of the OLED (V_TH_OLED_) is 1 V, and the equivalent capacitance of the OLED (C_OLED_) is 0.1 pF. Figure 1b shows the operation timing diagram of the switching signals. Three operation periods, including reset, programming, and emission, are proposed and described clearly, as presented in Figure 2.

### 2.1. Reset

In the reset period, the signal voltage of S_N-1_ is high to make T1 turn on. The power voltage, V_DD_ (5 V), is provided to Node G. S_n_ and E_m-1_ are kept low to make T2 and T3 turn off. Thus, there is no current flowing through the OLED, thereby preventing the flicker phenomenon.

### 2.2. Programming

During the programming period, the voltages of S_n-1_ and E_m_ are kept low to make T1, T4, and T6 turn off, and the voltages of S_n_ and E_m-1_ are kept high to make T2, T3, T5, and T7 turn on. Thus, the voltage of node G (V_G_) will discharge until the driving TFT (T7) turns off to make the threshold voltage of the driving TFT and the data voltage store in C_ST_. Then, the voltage of C_ST_ will become “V_DATA_ + V_TH_T7_”.

### 2.3. Emission

During the emission period, the voltages of S_n_ and S_n-1_ are kept low to make T1, T2, and T3 turn off, and the voltages of E_m_ and E_m-1_ are kept high to make T4, T5, T6, and T7 turn on. The function of OLED current (I_OLED_) is expressed as [19]

(1) $$I_{\mathrm{OLED}}=\frac{1}{2}k{\left(V_{\mathrm{GS}}-V_{\mathrm{TH}\_T7}\right)}^{2}\;=\frac{1}{2}k{\left(V_{\mathrm{DATA}}+V_{\mathrm{TH}\_T7}-V_{\mathrm{TH}\_T7}\right)}^{2}\;=\frac{1}{2}k{\left(V_{\mathrm{DATA}}\right)}^{2}$$

where k is μ × C_OX_ × *W/L*. Based on (1), the OLED current is independent of the threshold voltage of T7 (V_TH_T7_). Thus, threshold voltage variation of the driving TFT (ΔV_TH_DTFT_) is compensated by the proposed pixel circuit. Moreover, the use of the LTPS TFT enables the pixel circuit to supply sufficient driving current with a low driving voltage. Furthermore, the use of oxide TFTs makes the pixel circuit able to operate at a low frame rate, because it protects the storage voltage from variation. Thus, the proposed circuit is believed to effectively compensated for ΔV_TH_DTFT_ and can be operated at a low driving voltage with a low frame rate.

## 3. Results and Discussion

**Figure 3:**
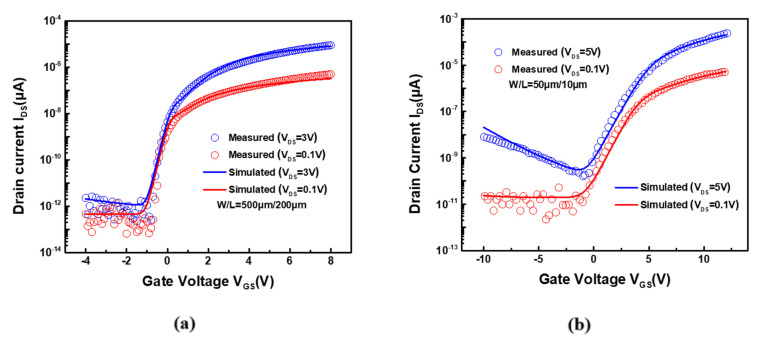
Simulated and measured transfer characteristics of the (**a**) a-IZTO TFT; (**b**) LTPS TFT.

To verify the operability of the presented compensation pixel circuit, the AIM-SPICE was used for simulation, and the transfer characteristics of the fabricated a-IZTO and LTPS TFTs were measured and fitted, as demonstrated in Figure 3a,b, respectively. Herein, HD (1280 × 720) displays at a frame rate of 60 Hz, the frame time is 16.6 ms, and the programming time for data input in each row was set to 23 μs.

**Figure 4:**
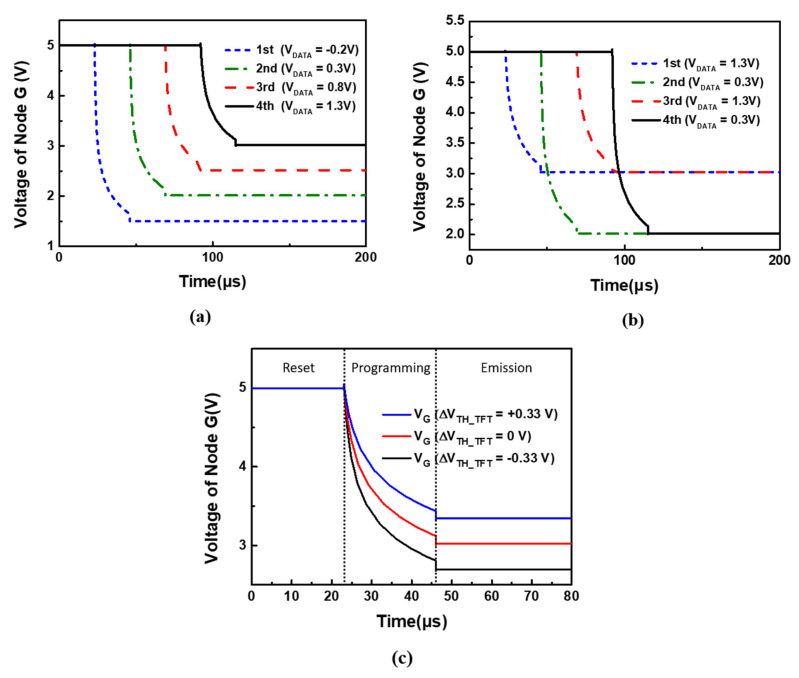
(**a**) Transient waveforms of V_G_ with first row to fourth row in different V_DATA_. (**b**) Transient waveforms of V_G_ with V_DATA_ = 1.3 V for first row and third row, V_DATA_ = 0.3V for second row and fourth row. (**c**) Transient waveforms of V_G_ for the presented compensation pixel circuit with ± 0.33 V variation in V_TH_DTFT_.

Figure 4a shows plots of the simulated transient waveforms of Node G (V_G_) in the pixels of the first row to the fourth row at different data voltages of −0.2, 0.3, 0.8, and 1.3 V respectively, to confirm that V_G_ receives the data correctly. Figure 4b shows the simulated transient waveforms of V_G_ with the same data voltage of 1.3 V applied to the pixels of the first row and third row, whereas the other data voltage of 0.3 V is applied to the pixels in the second row and fourth row. As shown in Figure 4b, the same voltage is obtained in a third row after the programming period, and the same result is presented for the second and fourth rows. Thus, Figure 4a,b show that the presented compensation pixel circuit receives data voltage successfully. Figure 4c demonstrates the waveforms of Node G for the presented 7T1C compensation pixel circuit when the V_TH_DTFT_ variation was ±0.33 V. As shown in Figure 4c, the V_TH_DTFT_ variation was sensed by the presented compensation pixel circuit.

**Figure 5:**
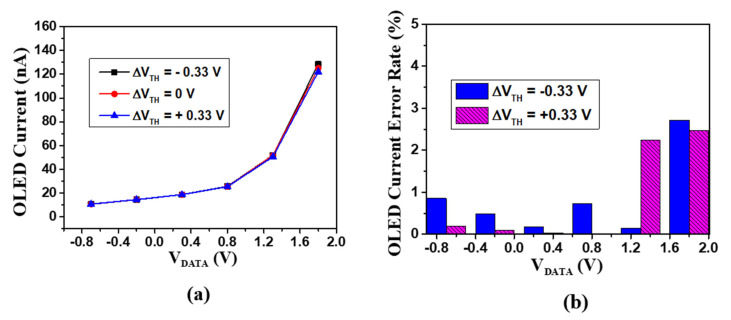
(**a**) OLED currents versus data voltage with ±0.33 V variation in V_TH_DTFT_. (**b**) OLED current error rates with ±0.33 V variation in V_TH_DTFT_.

Figure 5a shows plots of OLED current with different data voltages when the V_TH_DTFT_ variation was ±0.33 V. Figure 5b shows the relative current error rates over the range of data voltages with ΔV_TH_DTFT_ = ± 0.33 V. All error rates were lower than 2.71% within all gray levels, indicating excellent ability for compensating the V_TH_DTFT_ variation.

**Figure 6:**
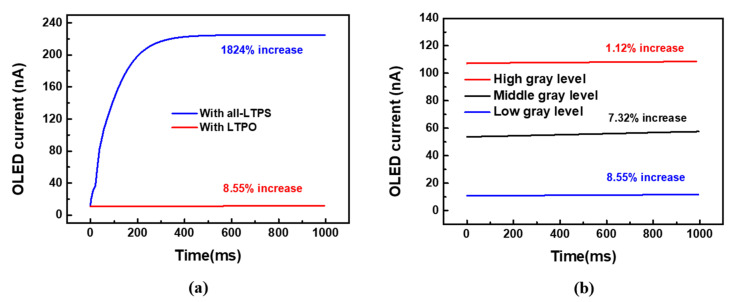
(**a**) OLED current variation with all-LTPS TFT and LTPO pixel circuit for 1 Hz frame rate (**b**) OLED current of high, middle, and low gray levels when the circuit is driven at 1 Hz.

Figure 6a shows a comparison of the OLED current variation between all-LTPS TFT and LTPO pixel circuits for a frame rate of 1 Hz. The LTPO can successfully protect the OLED current from variation. Figure 6b shows the OLED current of high, middle, and low gray levels when the circuit is driven at 1 Hz. The corresponding current variations were 1.12%, 7.32%, and 8.55% respectively, and they revealed the good ability of the present LTPO pixel circuit for maintaining the OLED current. Moreover, the features of the present compensation pixel circuit are listed and compared with the reported AMOLED compensation pixel circuits in Table 1. Based on the proposed pixel structure with two in-pixel signal lines, the subpixel layout area is estimated to be 42 μm × 21 μm, and is suitable for portable displays. Moreover, it shows that the error rate of 2.71% in the study is better than that of the reference [13] and is comparable to the references [15,16]. It was also found that the error rate is increased with the increasing ΔV_TH_DTFT_, because more compensation time would be needed for our pixel to achieve the varied V_TH_DTFT_. Although these reported circuits [13,14,15,16] could compensate for the ΔV_TH_DTFT_, only the presented pixel circuit has the ability to simultaneously achieve low-voltage driving and low frame rate, with only two signal lines including SN and EM in a pixel. Thus, the presented pixel circuit using an LTPO TFT is suitable to compensate for the ΔV_TH_DTFT_ for high-pixel-density portable AMOLED displays.

## 4. Conclusions

A new low driving voltage (5 V) compensation pixel circuit using LTPO TFT structure was presented for use in high-pixel-density portable AMOLED displays. We confirmed that the nonuniform OLED current caused by threshold voltage variation of driving TFT could be effectively compensated by the presented pixel circuit. The compensated error rates of OLED current were suppressed below 2.71% over the range of all gray levels when V_TH_DTFT_ variation was ±0.33 V. Moreover, a low frame rate of 1 Hz was achieved by the presented pixel circuit with no noticeable flicker, making it suitable for low power consumption applications. Accordingly, we believe that the presented compensation pixel circuit using LTPO TFTs is highly suitable for high-pixel-density portable AMOLED displays.

## Figures and Tables

**Table 1:** Comparison of proposed and prior pixel circuits.

Ref	This Study	[13]	[14]	[15]	[16]
**Signal lines**	2	2	4	3	4
** V ** **_TH_DTFT_** **variation error rate**	2.71% (ΔV_TH_ = ±0.33 V)	14.11% (ΔV_TH_ = ±0.5 V)	Null	4.73% (ΔV_TH_ = ±0.5 V)	2.8% (ΔV_TH_ = ±0.33 V)
**Driving voltage** **(V_DD_ & V_SS_)**	V_DD_ = 5 V V_SS_ = 0 V	V_DD_ = 11 V V_SS_ = 0 V	Null	V_DD_ = 3.3 V V_SS_ = −3.3 V	V_DD_ = 12 V V_SS_ = 0 V
**Backplane technology**	LTPO	LTPS	LTPO	LTPS	LTPS
**Minimum** **frame rate**	1 Hz	60 Hz	1 Hz	15 Hz	240 Hz
